# Overexpression of RACGAP1 by E2F1 Promotes Neuroendocrine Differentiation of Prostate Cancer by Stabilizing EZH2 Expression

**DOI:** 10.14336/AD.2023.0202

**Published:** 2023-11-09

**Authors:** Zhengshuai Song, Qi Cao, Bin Guo, Ye Zhao, Xuechao Li, Ning Lou, Chenxi Zhu, Gang Luo, Song Peng, Guohao Li, Ke Chen, Yong Wang, Hailong Ruan, Yonglian Guo

**Affiliations:** ^1^Department of Urology, The Central Hospital of Wuhan, Tongji Medical College, Huazhong University of Science and Technology, Wuhan, China; ^2^Department of Urology, Union Hospital, Tongji Medical College, Huazhong University of Science and Technology, Wuhan 430022, China; ^3^Cancer Center, Union Hospital, Tongji Medical College, Huazhong University of Science and Technology, Wuhan 430022, China; ^4^Department of Urology, Tongji Hospital, Tongji Medical College, Huazhong University of Science and Technology, Wuhan, 430030, China; ^5^Hubei Institute of Urology, Wuhan 430030, China

**Keywords:** RACGAP1, EZH2, E2F1, ubiquitin, neuroendocrine prostate cancer (NEPC)

## Abstract

Neuroendocrine prostate cancer (NEPC) is a lethal subtype of prostate cancer. It is characterized by the loss of androgen receptor (AR) signaling in neuroendocrine transdifferentiation, and finally, resistance to AR-targeted therapy. With the application of a new generation of potent AR inhibitors, the incidence of NEPC is gradually increasing. The molecular mechanism of neuroendocrine differentiation (NED) after androgen deprivation therapy (ADT) remains largely unclear. In this study, using NEPC-related genome sequencing database analyses, we screened *RACGAP1*, a common differentially expressed gene. We investigated RACGAP1 expression in clinical prostate cancer specimens by IHC. Regulated pathways were examined by Western blotting, qRT-PCR, luciferase reporter, chromatin immunoprecipitation, and immunoprecipitation assays. The corresponding function of RACGAP1 in prostate cancer was analyzed by CCK-8 and Transwell assays. The changes of neuroendocrine markers and AR expression in C4-2-R and C4-2B-R cells were detected in vitro. We confirmed that RACGAP1 contributed to NE transdifferentiation of prostate cancer. Patients with high tumor RACGAP1 expression had shorter relapse-free survival time. The expression of RACGAP1 was induced by E2F1. RACGAP1 promoted neuroendocrine transdifferentiation of prostate cancer by stabilizing EZH2 expression in the ubiquitin-proteasome pathway. Moreover, overexpression of RACGAP1 promoted enzalutamide resistance of castration-resistant prostate cancer (CRPC) cells. Our results showed that the upregulation of RACGAP1 by E2F1 increased EZH2 expression, which drove NEPC progression. This study explored the molecular mechanism of NED and may provide novel methods and ideas for targeted therapy of NEPC.

## INTRODUCTION

Prostate cancer (PCa) is the most common male malignant tumor worldwide, ranking as the third leading cause of cancer-related deaths in men [1]. Metastatic PCa can develop into castration-resistant prostate cancer (CRPC) after androgen deprivation therapy (ADT). At present, a new generation of powerful androgen receptor (AR) inhibitors, such as enzalutamide, can alleviate CRPC progression, but many patients will eventually develop drug resistance. Moreover, due to the extensive use of these drugs, the occurrence of an AR-independent, more malignant and lethal neuroendocrine prostate cancer (NEPC) is rapidly increasing, accounting for 25% of metastatic CRPC patients [2]. Patients with NEPC have a local invasion or distant metastasis, and the prognosis is very poor with a survival time of less than 1 year. At present, the biological behavior of NEPC is not well explored, the mechanism of neuroendocrine transdifferentiation (NED) in CRPC is not clear, and the clinical treatment for NEPC is limited [3, 4].

Current studies have shown that the differentiation of CRPC to NEPC is related to many molecular mechanisms, such as mutations of the tumor suppressor genes *RB1*/*E2F1* and *P53* [5] [6], an increase in mitosis through various genes such as *AURKA* and *PEG10* [7], amplification of the *MYCN* gene, and changes in the splicing factor *SSRM4* and epigenetic genes *REST*, *SOX2*, and *EZH2* [8-11]. At present, the increase in neuroendocrine differentiation markers (such as *CHGA* and *SYP*) is the main indicator of neuroendocrine differentiation in PCa [12].

RacGTP enzyme activating protein 1 (RACGAP1), also known as MgcRacGAP, is a GTP enzyme activating protein family member. Previous studies have confirmed that RACGAP1 promoted proliferation, differentiation, and metastasis of many malignant tumors, and potentially, it is a prognostic indicator [13-16]. A previous study showed that RACGAP1 regulated the downstream factors of the PI3K/AKT signaling pathway and that the compensatory activation of the PI3K/AKT signaling pathway was closely associated with ADT drug resistance and neuroendocrine differentiation in PCa [13]. In addition, RACGAP1 activated the STAT3-survivin signaling pathway in uterine sarcoma and hepatocellular carcinoma [14, 15]. STAT3 promoted the neuroendocrine differentiation of PCa mediated by EZH2 and IL6 [16]. Another study showed a direct positive correlation between RACGAP1 and AURKA in gastric cancer and that AURKA is also an important neuroendocrine (NE) marker of PCa [17]. However, the role of RACGAP1 in PCa and neuroendocrine transformation has not been reported.

The histone methyltransferase enhancer of zeste homolog 2 (EZH2) inhibits the transcription of downstream target genes mainly by methylation of H3K27, and it has been shown to be significantly overexpressed in NEPC clinical samples compared to PCa samples[18]. Recent studies showed that EZH2 altered the epigenetic programming in NEPC cells and promoted NEPC cell growth [6, 8, 10]. Another study found that GSK503, which silenced and inhibited EZH2, restored the sensitivity of *PTEN* and *RB1* double knockdown mice to enzalutamide [6]. However, the detailed mechanisms underlying the increased EZH2 still need to be further elucidated.

In this study, we found RACGAP1 as a potential master regulator of NED. The high expression of RACGAP1 in NEPC tissues was positively correlated with the stage and grade of the tumor and suggested a poor prognosis of patients. In NEPC, the increase of E2F1 promoted the upregulation of *RACGAP1* expression. RACGAP1 then promoted NED in PCa by upregulating the expression of EZH2, which is the key target of neuroendocrine transformation in PCa. This study provides deep insight into the molecular mechanism of NEPC and provides novel methods and ideas for targeted therapy of NEPC.

## MATERIALS AND METHODS

### Cell culture and reagents

LNCaP, NCI-H660, C4-2, DU145, 22RV1, RWPE-1, 293T and PC3 cell lines were obtained from the American Type Culture Collection (ATCC). RWPE-1 cells were maintained in K-SFM medium containing bovine pituitary extract and human epidermal growth factor at 37°C and 5% CO_2_. Other prostate cancer cells were cultured in RPMI-1640 medium containing 1% penicillin and 10% fetal bovine serum (FBS). LNCaP cells were cultured in RPMI-1640 medium containing 5% activated carbon/dextran-treated FBS. 293T cells were cultured in DMEM. Drug concentrations (unless otherwise indicated) were dihydrotestosterone (DHT) (10 nM), enzalutamide (20 µM), chloroquine (50 μM) or MG132 (20 μM), and cycloheximide (10 µM). These drugs were all obtained from MCE (China) Medchemexpress Co., Ltd.

### Human samples

We collected 10 pairs of normal adjacent tissues and PCa tissues and seven cases of prostate cancer tissues with obvious expression of neuroendocrine markers from the Department of Urology, Wuhan Union Hospital, between 2019 and 2021. Excised tissues were rapidly collected and stored in liquid nitrogen. Before surgery, the patients had not received any hormone therapy, radiotherapy, chemotherapy, or immunotherapy. These studies were conducted in accordance with approved guidelines. All experiments were approved by the Human Ethics Committee of Huazhong University of Science and Technology ([2020] IEC-J475).

### Establishment of the NE-like cell line C4-2B-N

The initial dose of enzalutamide in the parental C4-2 and C4-2B cell culture medium was 0.2 mmol/L. At this concentration, the cells were stably divided into three generations. The drug dose was then improved, and the culture was continued. As our lab described previously, the concentration gradients of enzalutamide were 0.4, 0.8, 1, 2, 4, 8, 10, 20, and 40 mmol/L[19]. In brief, by adding enzalutamide (induction time > 6 months) to parental C4-2 and C4-2B cell culture medium with continuous low-dose induction and intermittent high-dose shock, an NE-like cell line named C4-2B-N was successfully constructed.

### Bioinformatics analysis

The appropriate PCa data were downloaded from the Gene Expression Omnibus (GEO; www.ncbi.nlm.nih.gov/geo/) database, and data were also obtained from Beltran, 2016 [20], YAN HU et al.[21], MSKCC, Cancer Cell 2010 Taylor [22], and MCTP, Nature 2012 Grasso [23]. The differential genes in each database were analyzed according to logFC ≥ 2 and p < 0.05. According to the changes of *RACGAP1* and NE marker mRNAs in the NEPC database (Beltran, 2016) [32], a heat map was drawn, and the correlation was analyzed. The relationship between the expression of *RACGAP1* mRNA and the progression and prognosis of patients with PCa was analyzed according to the *RACGAP1* mRNA expression in data from TCGA-PRAD obtained from the cBioPortal (http://www.cbioportal.org/public-porta) and Cancer Cell 2010 Taylor [22]. Data samples from Beltran, 2016 were divided into two groups (high versus low) based on the mRNA expression level of *RACGAP1*, and the median expression data were used as the cut-off point. Gene Set Enrichment Analysis (GSEA) (http://software.broadinstitute.org/gsea/index.jsp) was performed on the two groups to explore the potential effect of *RACGAP1*.

### Lentivirus, plasmids, and shRNA

Lentiviral particles containing GV492-FLAG-RACGAP1 (catalog no. GXDL0174171) were purchased from Shanghai GeneChem (China) and added to cells with polybrene to overexpress *RACGAP1* based on the manufacturer’s recommendations. Small hairpin RNA (shRNA) against human *RACGAP1* (catalog no. GIEE0172851) and the corresponding control with nonsense sequences were obtained from Shanghai GeneChem. The shRNA sequences targeting *EZH2* were annealed and cloned into the AgeI and EcoRI sites of the plko.l plasmid (Addgene). The shEZH2 sequence was 5’-GGACGGCTCCTCTAACCATGT-3’. The siRNA sequences targeting E2F1 were purchased from Guangzhou RiboBio Co., Ltd. The sequences were siE2F1-1: 5’-TGGACCACCTGATGAATAT-3’ and siE2F1-2: 5’-GAGGAGTTCATCAGCCTTT-3’. Expression plasmids for GFP-EZH2 and Myc-ubi were also obtained from Genechem. In general, 2 µg of the plasmid was mixed with Lipofectamine® 2000 (Invitrogen) and then added to the cells for transfection.

### Lentiviral constructs

The cell culture medium was replaced with serum-free H-DMEM 4 h before transfection. The cells were incubated at room temperature for 5 min with a mixture of 500 µL of opti-MEM and Lipofectamine 2000. Then, 500 µL of Opti-MEM, lentivirus vector, pMD2.G, pRRE, and pRSV/REV was mixed to obtain DNA mixed suspensions. Then, the DNA suspensions were mixed with Opti-MEM and Lipofectamine 2000, incubated for 20 min, and added to the target cell culture medium. After 6 h, the medium was replaced with a complete medium containing serum. The cell supernatant was collected at 48 h and 72 h, filtered using a 0.45-µm filter membrane, and added to the cell culture medium. Screening of transfected cells using puromycin and western blotting was performed to verify the protein expression level.

### Immunohistochemistry

After the tissues were fixed using 4% paraformaldehyde, the sliced tissues were embedded in paraffin wax and dewaxed. After antigen retrieval and blocking endogenous peroxidase, the cells were blocked with BSA for about 30 min and then incubated overnight in a humidified chamber at 4°C after incubating with RACGAP1 (1:200, ab134972, Abcam, USA) and Ki67 (1:10000, 27309-1-AP, Proteintech, China) antibodies. Then, the slices were washed for 5 min with PBS three times. After drying, secondary antibodies (1:200; GB23303; Servicebio, Inc., Woburn, MA, USA) corresponding to the primary antibody were added to the tissue slices and incubated at room temperature for 2 h. Images were acquired, and the IHC data were analyzed by two blinded pathologists. The immunoreactivity was evaluated by two investigators using immunoreactivity scores, which ranged from 0 to 12, based on the staining intensity score (0-3) and the positive cell proportion score (0-4). IRS scores of 0-1 was considered negative; scores of 2-3 were considered mild; scores of 4-8 were considered moderate; and scores of 9-12 were considered strongly positive.

### Chromatin co-immunoprecipitation (ChIP) assays

ChIP assays were performed as previously described [19]. In brief, the ChIP assay was conducted according to the protocol of the ChIP assay kit (CST). C4-2 and PC3 cells were cultured in 10-cm dishes. Then, 243 µL of 37% formaldehyde was added to one plate to a final concentration of 1% (the culture medium had a total of 9 mL). After incubating at 37°C for 10 min, the chromatin was cross-linked, followed by rinsing twice with 2 mL of precooled PBS including protease inhibitors. The cells were then lysed in ChIP lysis buffer and sonicated to entirely disrupt the nuclei. The ChIP buffer was used to dilute the digested, cross-linked chromatin. A 10 µL sample was extracted from the diluted chromatin as a 2% input group. Then, 500 µL of the remaining diluted chromatin and corresponding antibodies (anti-E2F1, ProteinTech, catalog no. 66515-1-Ig, or anti-IgG, Beyotime Institute of Biotechnology, catalog no. A7001) were incubated overnight at 4°C with rotation. Following elution of chromatin, reversion of cross-links, and DNA depuration, qRT-PCR was carried out to identify the potential E2F1 binding sites on the RACGAP1 promoter region.

### Immunoprecipitation and western blot

Cells were collected, added to an appropriate amount of cell immunoprecipitation cleavage buffer (including protease inhibitors), lysed on ice or at 4°C for 30 min, and centrifuged at 12,000 g for 30 min. A small amount of lysate was used for Western blot analysis, and 1 μg of the corresponding antibody and 10-50 μL of protein A/G-beads were added to the remaining cell lysate and rotated slowly at 4°C in an overnight incubation. The next day, the protein A/G-beads were centrifuged at 3000 g for 5 min at 4°C. The supernatant was carefully discarded, and the protein A/G-beads were washed with 1 mL of lysis buffer 3-4 times. Finally, 15 μL of 2 × SDS buffer was added and the samples were boiled for 10 min. For western blotting analysis, total proteins were analyzed by 10% SDS-PAGE and transferred to polyvinylidene fluoride (PVDF) membranes. The PVDF membranes were blocked and infiltrated with corresponding primary antibodies. Western blotting was performed as described previously [24]. The primary antibodies included RACGAP1 (1:1000, ab134972 Abcam, USA), E2F1 (1:3000, 66515-1-Ig, Proteintech, China), CHGA (1:1000, ab283265, Abcam, USA), SYP (1:1000, ab32127, Abcam, USA), ENO2 (1:1000, ab79757, Abcam, USA), AR (sc-7305; Santa Cruz Biotechnology), PSA (1:3000, 10679-1-AP, Proteintech, China), EZH2 (1:1000, 21800-1-AP, Proteintech, China), FLAG (1:5000, 20543-1-AP, Proteintech, China), MYC (1:5000, 60003-2-Ig; Proteintech, China), β-actin (1:3000, 20536-1-AP, Proteintech, China), and GAPDH (1:3000, 10494-1-AP, Proteintech, China). The membranes were incubated using horseradish peroxidase-conjugated secondary antibodies (1:3000, SA00001-2, Proteintech, China) and then exposed to an enhanced chemiluminescence substrate (Thermo, Massachusetts, USA).

### Cell proliferation assay

A total of 3×10^3^ cells were seeded per well of a 96-well plate. A Cell Counting kit-8 (CCK-8) (DOJINDO Laboratories, Kumamoto, Japan) was used to detect cell proliferation every 24 h. Briefly, a solution containing 10 µL of CCK-8 was added per well. After 2 h, the absorbance was detected at 450 nm using a spectrometer. Cell viability was measured after 24, 48, 72, and 96 h based on the relative optical density. All experiments were performed in triplicate.

### Transwell assay

The migration, and invasion of cells were analyzed according to previously published cell growth, Transwell migration, and Matrigel invasion experiments [25]. First, 24-well Transwell chambers with 8-µm pore membranes (Corning LifeSciences, cat. no. 353097) were used to detect cell migration and invasion. The Transwell chamber utilized for invasion assays was pre-coated with Matrigel (BD Biosciences, San Jose, CA, USA). Prostate cancer cells (20,000) were incubated in serum-free medium for 24 h and then seeded onto the upper chamber of the Transwell insert, and media containing 10% FBS were placed in the bottom chamber as a chemoattractant. The chambers were cultured at 37°C in a cell incubator for 48 h, washed twice carefully with PBS, and then fixed in 100% methanol at a low temperature for 15 min. Then, the cells were stained with 0.05% crystal violet at room temperature, and five fields were randomly chosen for analysis. All experiments were performed in triplicate.

### RNA isolation and RT-PCR

Total RNA from collected cells was extracted using TRIzol reagent (Thermo Scientific, catalog no. AM9738) according to the manufacturer’s descriptions. The concentration and purity of the RNA solution were measured by a NanoDrop 2000 spectrophotometer (NanoDrop Technologies, Wilmington). Using 0.5 µg of cell RNA, first-strand cDNA synthesis was performed according to the recommendations of the RevertAid First Strand cDNA Synthesis kit (TaKaRa, catalog no. RR014A). Real-time PCR was performed using Maxima SYBR Green/ROX qPCR Master Mix (MBI Fermentas) on an ABI-7500 qRT-PCR analysis system. The relative mRNA expression of genes was quantified using the power formula: 2 ^(-ΔΔCt). qRT-PCR was executed on a Roche LightCycler 480 system. Levels of target genes were normalized to *GAPDH* expression. All experiments were performed in triplicate. The primer sequences applied were as follows: *RACGAP1* (forward, 5’-TGGG AAGTAACAGGCAGA-3’; reverse, 5’-ATGATGGTG G AGCAAGAG-3’), *EZH2* (forward, 5’-TGAGGCTTC AGCACCACT-3’; reverse, 5’-ACGGCTTCCCAATAA CAG-3’), *E2F1* (forward, 5’-CAGGGTCTGCAATGC TACGA-3’; reverse, 5’-TGAACTGGGCTGCCGAGGT G-3’), *NCAM1* (forward, 5’-CGGAGGCTTCACAGGT AA-3’; reverse, 5’-GTGGATAAGAACGACGAGG-3’), *NSE* (forward, 5’-AACTCTGAGGCAGCAACAT-3’; reverse, 5’-AGGACAAATACGGCAAGG-3’), *SYP* (forward, 5’-TGGAGTAGAGGAAGGCAAACA-3’; reverse, 5’-CGGACATGGACGTGGTGA-3’), *CHGA* (forward, 5’-TGGAGGGTGGGTGTTGGT-3’; reverse, 5’-GCTGAAAGAGGCGGTGGA-3’), *GAPDH* (forward, 5’-TCAAGAAGGTGGTGAAGCAG-3’; reverse, 5’-CGTCAAAGGTGGAGGAGTG-3’).

**Figure 1.**
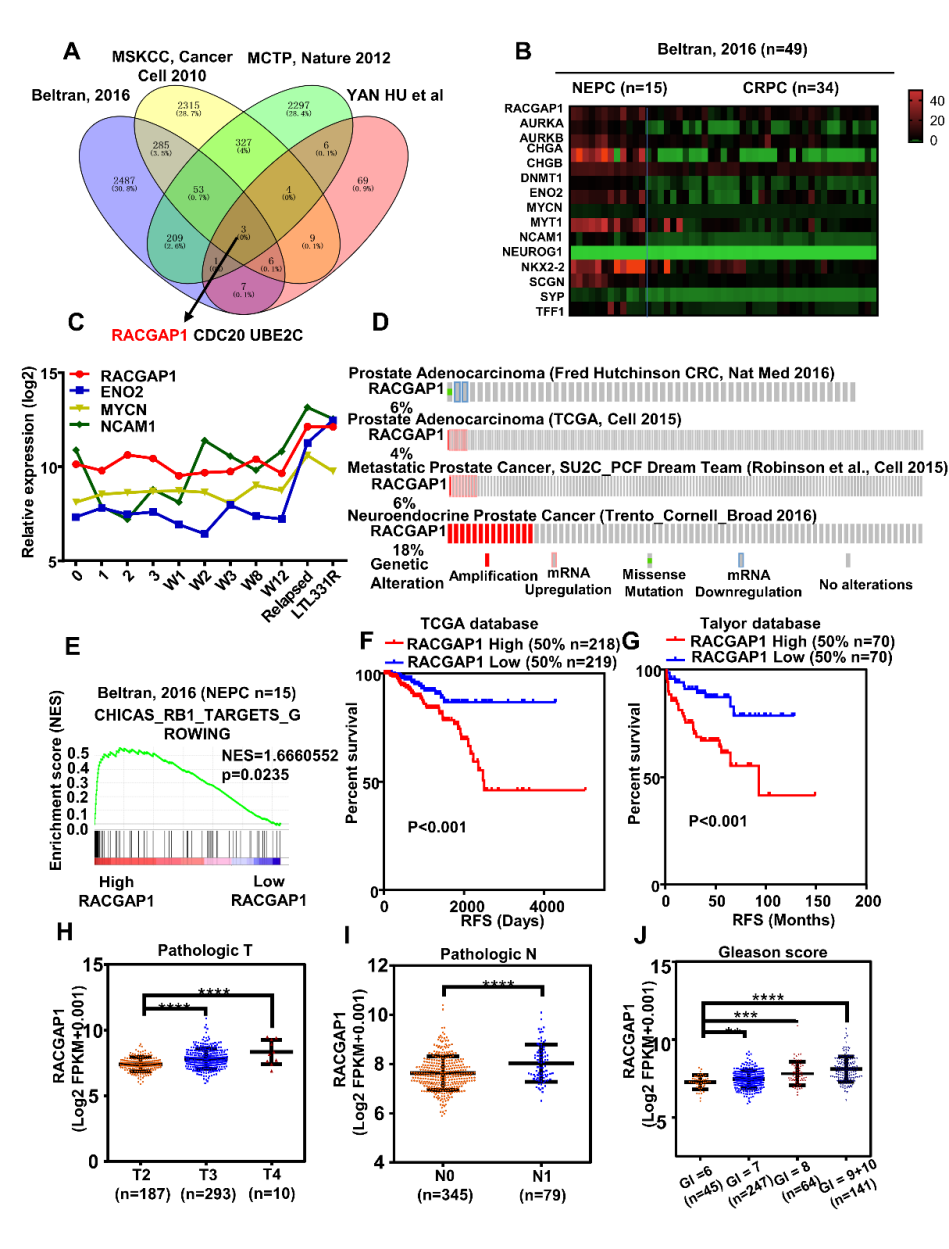
Expression of RACGAP1 was increased in NEPC and was related to the progression and prognosis of prostate cancer. (A) Venn diagram showing that three highly expressed genes were screened from four NEPC-related public databases. (B) Heatmap showing the mRNA expression of RACGAP1 and NED markers in NEPC (n=15) and CRPC tumor tissues (n=34) of prostate in Beltran, 2016 (n=49) database. (C, D) Other visual analyses of multiple databases showed that the mRNA expression of *RACGAP1* in NEPC was significantly higher than that in other types of prostate cancer and was positively correlated with NED markers (adenocarcinoma pre-castration day 0; post-castration days 1-3; weeks 1-3, 8, and 12; and post-NEPC development relapsed and LTL331R. Data were downloaded from GSE59986). (E) High expression of *RACGAP1* in NEPC (n=15) was enriched in the RB1 signaling pathway. (F, G) Kaplan-Meier analysis showed that poor prognosis with high *RACGAP1* of recurrence-free survival (RFS) of data in the TCGA-PRAD (n=437) and Taylor databases (n=140). (H-J) mRNA levels of *RACGAP1* in prostate cancer in the TCGA-PRAD database were related to different clinicopathological parameters: pathological T stage (n=490), pathological N stage (n=424), and Gleason score (n=497). TCGA-PRAD, The Cancer Genome Atlas Prostate Cancer. ***, p < 0.001, **, p < 0.01, *, p < 0.05.

### Luciferase assays

Cells were cultured on a 24-well plate at 70% confluence, and 0.8 µg of expression vector plasmids and 0.4 µg of promoter-reporter plasmids were used for transient transfection. The fluorescence value was detected after 48 h. The Renilla luciferase activity, a standard internal control, was used to normalize the reporter luciferase activity of the gene promoter. The plasmids of RACGAP1-luc (catalog no. GOSE0200448) and mutated RACGAP1-luc (catalog no. GOSE0162288) were designed and constructed by Genechem (Shanghai).

### Tumor xenograft study

All animal procedures were approved by the Animal Ethics Committee of Tongji Medical College of Huazhong University of Science and Technology. A total of 40 nude mice were used in the animal experiment. Each group contained five mice. The castrated male BALB/c nude mice aged 3-4 weeks were purchased from Beijing Weishenghe Experimental Animal Science and Technology Co., Ltd. After cultured cells grew to a logarithmic phase, the cells were digested by trypsin and a cell suspension was prepared (the cell density was 1 × 10^6^/100 µL). Then, 100 µL of the cell suspension was subcutaneously injected into the axilla of nude mice to establish a model of a subcutaneously transplanted tumor. An in vivo metastasis model was established by intravenous injection of 2 ×10^6^ cells through the tail vein. The growth of tumors was observed every 3 days. The tumor volume (V) was calculated as follows: V = length (L) × width (W)^2^/2, every 5 days after developing tumors, and the mice were killed 60 days later.

### Statistical analysis

All statistical analyses were performed using Prism 6.0 (GraphPad) and SPSS 22.0 software (IBM Corporation). All in vitro assays were repeated three times. Normally distributed continuous variables were indicated as the mean ± SD, while non-normally distributed continuous variables were indicated by the median. For normally distributed variables, two experimental groups were analyzed by independent Student’s *t* test. One-way ANOVA, followed by Tukey’s multiple comparison test, was applied to compare multiple groups. The Pearson correlation test was utilized to determine the correlation between normal distribution variables, whereas a Spearman correlation test was used to detect non-normal distribution variables. Survival status was assessed by the Kaplan-Meier method and examined using the log-rank test. P < 0.05 was statistically significant.

## RESULTS

### RACGAP1 is a potential regulator of neuroendocrine transdifferentiation

Given the close relationship between PCa metastasis and NE transformation, we screened the different genes in two neuroendocrine PCa databases and two large PCa metastasis databases (logFC ≥ 1.5, p < 0.05). Three common genes (*RACGAP1*, *CDC20*, and *UBE2C*) that could be used as candidate genes were identified (Fig. 1A). A previous study reported that the expression of *RACGAP1* in NEPC was significantly higher than that in PCa of transgenic mice [21], but *CDC20* and *UBE2C* in NEPC have not been reported. Furthermore, the difference in *RACGAP1* expression and the correlation between *RACGAP1* and NE markers were intuitively analyzed using a heat map of the NEPC database [20]. The results showed that *RACGAP1* in NEPC was significantly overexpressed and positively correlated with NE markers (Fig. 1B). Then, the detailed degree of correlation was further analyzed, and we found a significant positive relationship between *RACGAP1* and NE markers, while the other two genes (*CDC20* and *UBE2C*) had no significant correlation with the NE markers *CHGA* and *ENO2* (Supplementary Fig. 1A, B). In another NEPC mouse PDX model, the expression of *RACGAP1* and related NE markers in different periods after castration, recurrence, and NEPC was analyzed, and we found that the expression of *RACGAP1* increased with NE markers in NEPC recurrence (Fig. 1C). Analysis of different PCa databases showed that the amplification of *RACGAP1* became more notable as the disease progressed, and the increase was the most significant in NEPC (Fig. 1D). Based on data from literature and bioinformatics analysis, we chose *RACGAP1* as the gene for further study. We also analyzed the relationship between *RACGAP1* and other neuroendocrine genes (Supplementary Fig. 1B). GSEA of the gene probe enrichment in the Beltran, 2016 database showed that the upregulated expression of *RACGAP1* was closely related to RB1_TARGETS_GROWING, a key genetic event of NED (Fig. 1E). Analysis of the TCGA database and Taylor database [22] showed that high expression of *RACGAP1* was associated with poor relapse-free survival time (RFS) (Fig. 1F, G). Additionally, the relationship between *RACGAP1* and clinicopathological parameters was analyzed in TCGA. Our results showed that the expression level of *RACGAP1* increased with the increase of pathological stage, grade, metastasis, and Gleason score, suggesting that *RACGAP1* may promote the progression of PCa (Fig. 1H-J, Supplementary Fig. 2A, and Table 1). Taken together, these series of results suggested a potential role of *RACGAP1* in promoting the progression of PCa and that it may be a regulator of NED. However, its function in NED and NEPC is unknown.

**Table 1:** Correlation between RACGAP1 mRNA expression and clinicopathological parameters of prostate cancer patients.

			RACGAP1 mRNA expression		
Parameter		Number	Low (n=155)	High (n=154)	P value
Age(years)	< 60	116	63	53	
	≥ 60	193	92	101	0.291
PSA	< 4	297	151	146	
	≥ 4	12	4	8	0.257
T stage	T1 or T2	110	70	40	
	T3 or T4	199	85	114	0.001
N stage	N0	257	137	120	
	N1	52	18	34	0.015
M stage	M0	308	155	153	
	M1	1	0	1	0.498
Biochemical recurrence	NO	270	145	125	
	YES	39	10	29	0.001
Gleason score	<8	168	100	68	
	≥8	141	55	86	0.000

### Expression of RACGAP1 in NEPC promotes NED after enzalutamide treatment

**Figure 2.**
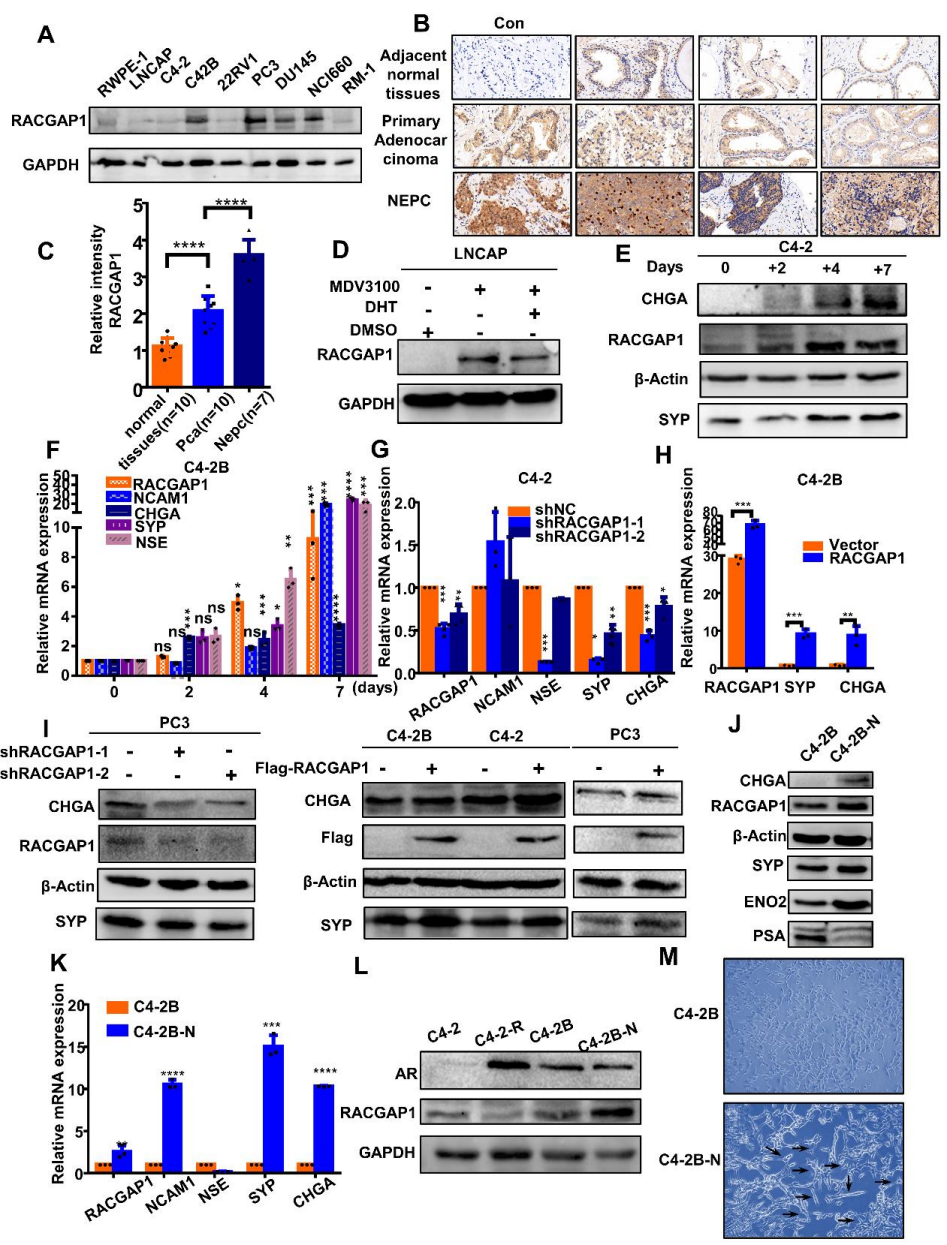
RACGAP1 overexpression promoted neuroendocrine transformation in prostate cancer. (A) Western blot analysis showed that RACGAP1 was upregulated in NEPC-like cells. (B) Immunohistochemical study of different types of prostate cancer tissues showed that RACGAP1 was highly expressed in NEPC. (C) Data for quantified immunohistochemistry in adjacent normal tissues (n=10), tumor tissues (n=10) and NEPC (n=7) of prostate cancer are shown as mean + SD. (D) Enzalutamide (MDV3100) induced the production of *RACGAP1*, and DHT partially reversed this effect. (E) Western blot analysis showed the protein expression of RACGAP1, CHGA, and SYP in cells treated with or without 10 μmol/L enzalutamide for 2, 4, or 7 days. (F) The mRNA level of *RACGAP1*, *NCAM*, *CHGA*, *SYP*, and *NSE* in cells treated with or without 10 μmol/L enzalutamide for 2, 4, or 7 days were determined by qRT-PCR analysis. (G) *RACGAP1* and NE markers (*CHGA*, *NCAM*, *NSE*, and *SYP*) in C4-2 cells following transient transfection with control (shNC) or *RACGAP1* shRNA (sh1, sh2), as detected by qRT-PCR. (H) *RACGAP1* and NE markers (*CHGA*, *SYP*) in C4-2B cells following transient transfection with RACGAP1 or an empty vector, as detected by qRT-PCR. (I) Protein expression of RACGAP1 and NE markers (CHGA, and SYP) in prostate cancer cells following transient transfection with control (shNC) or *RACGAP1* shRNA (sh1, sh2) and FLAG-RACGAP1 or an empty vector were determined by Western blot analysis. (J, K, L) Western blot and qRT-PCR were performed to detect protein and relative mRNA expression of *RACGAP1*, NE markers, and AR in NE-like cells (C4-2B-N). (M) Morphological changes of C4-2B-N compared with C4-2B cells under a microscope. Bar graphs show the statistical analysis of three independent experiments. ***, p < 0.001; **, p < 0.01; *, p < 0.05, p = ns (no significance); *t* test for two groups or ANOVA for more than two groups.

First, we examined the expression of *RACGAP1* in NEPC cells (NCI-H660) and tissues. We found that the expression of *RACGAP1* was significantly increased in NCI-H660 (NEPC) [26] cells, PC3 cells (NE-like cells), and DU145 (NE-like cells) [27-29], as compared to other cells, including RWPE-1, LNCAP, 22RV1, and C4-2 (Fig. 2A). We also found a higher expression of *RACGAP1* in NEPC tissues compared to primary PCa and adjacent normal tissues (Fig. 2B, C). Then, parental LNCAP cells were treated with 10 µmol/L enzalutamide or 10 µmol/L enzalutamide with 10 umol/L dihydrotestosterone (DHT) for 48 h, and the untreated parental LNCAP cells served as controls to detect the changes in *RACGAP1* protein expression. Our results showed that the expression of *RACGAP1* was significantly increased in the cells stimulated by enzalutamide but decreased again after adding DHT (Fig. 1D). Furthermore, with the increase of the stimulation time of enzalutamide, the expression of *RACGAP1* and NE markers showed a consistent increase (Fig. 2E, F). These findings showed that enzalutamide treatment induced the expression of *RACGAP1*, which is critical for ADT-induced NED of prostate cancer cells. Next, to further confirm the effect of *RACGAP1* on NED of PCa, we knocked down or overexpressed *RACGAP1* in different PCa cell lines to observe the expression of the NE markers *CHGA* and *SYP* at the mRNA and protein levels. The results showed that downregulation of *RACGAP1*, *CHGA*, and *SYP* significantly decreased. Overexpression of *RACGAP1* significantly increased the chances of the two markers (Fig. 2H, I). It has been further suggested that *RACGAP1* can promote the NED of prostate cancer. To efficiently conduct cell research, we detected the expression of neuroendocrine markers in C4-2-R and C4-2B-R cells, our enzalutamide-induced cell line [30], which showed that the expression of *RACGPA1* and neuroendocrine markers and the number of cells with nerve cell morphology were significantly increased, and there was lower AR expression in C4-2B-R cells (Fig. 2J-M, Supplementary Fig. 2B). This new cell line was named C4-2B-N.

### Effect of RACGAP1 on biological behavior of prostate cancer in vitro

**Figure 3.**
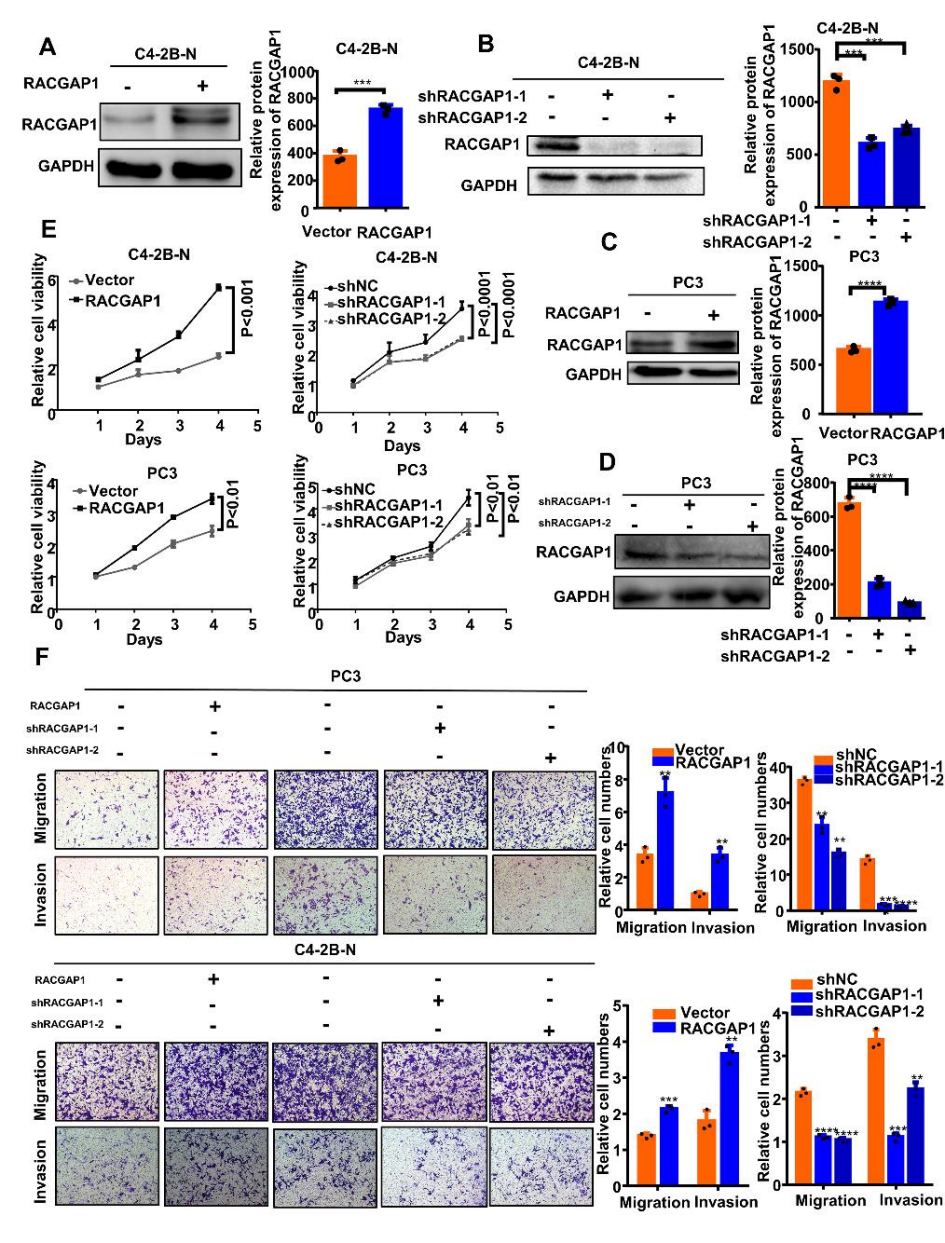
RACGAP1 increased proliferation, migration, and invasion of prostate cancer cells in vitro. (A-D) Western blotting and protein level analysis of *RACGAP1* upregulation and knockdown in C4-2B-N and PC3 cells; GAPDH was used as internal control. (E) Cell counting kit-8 assays were conducted to detect the effects of *RACGAP1* upregulation and knockdown on the proliferation of NE-like cells (C4-2B-N and PC3 cells). (F) Effects of *RACGAP1* upregulation and knockdown on NE-like cell migration and invasion were explored by Transwell assays (left panels). Comparative quantitative analysis of the number of NE-like cells that migrated or invaded the membrane between the experimental and control groups (right panels). Bar graphs show the statistical analysis of three independent experiments. ***, p < 0.001; **, p < 0.01; *, p < 0.05, p = ns (no significance); *t* test for two groups or ANOVA for more than two groups.

To study the effect of *RACGAP1* on the biological behavior of NEPC cells, we used shRNA that interfered with *RACGAP1* expression and *RACGAP1* plasmid to overexpress *RACGAP1* by transfecting C4-2B-N cells (NE-like cells) and PC3 cells. First, the effects of knockdown and overexpression of *RACGAP1* were detected by Western blotting (Fig. 3A-D). The results of the CCK-8 assay showed that the proliferation ability of overexpressed *RACGAP1* cells was significantly higher than that of the control group. In these two cell lines, with knockdown of *RACGAP1*, the proliferation ability was significantly decreased compared with the control group (Fig. 3E). Transwell assays showed that overexpression of *RACGAP1* significantly enhanced the invasion and migration ability of these cells, while knockdown of *RACGAP1* significantly inhibited their migration and invasion ability (Fig. 3F). These experiments confirmed that the change of *RACGAP1* expression significantly promoted the progression of PCa.

**Figure 4.**
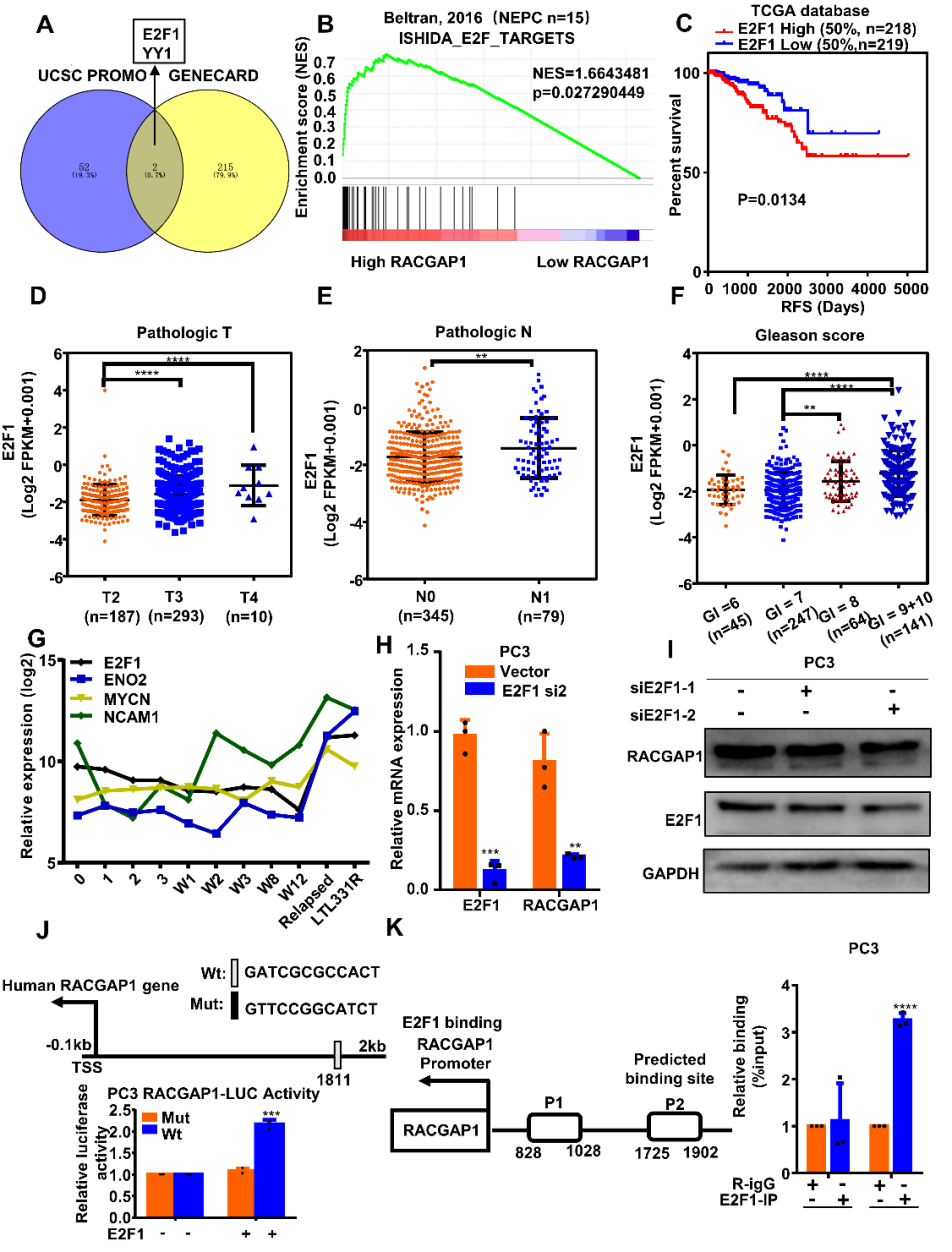
E2F1 transcriptional regulation of *RACGAP1* increase. (A) RACGAP1 transcription factor was predicted by the sequence 2000 kb upstream of the transcription initiation site of *RACGAP1* in UCSC-PROMO that intersected with the transcription factor sites predicted by GENEGARD. (B) GSEA was used to show that high *RACGAP1* expression was enriched in the downstream pathway of E2F1. (C) Kaplan-Meier curves showed that high expression of E2F1 predicted shorter RFS in TCGA-PRAD (n=437). (D-F) mRNA levels of *E2F1* in prostate cancer datasets downloaded from TCGA-PRAD were related to different clinicopathological parameters: pathological T stage (n=490), pathological N stage (n=424), and Gleason score (n=497). (G) Patient-derived xenograft model of terminally differentiated NEPC (LTL331R) showed that *E2F1* was significantly overexpressed (GSE59986). (H, I) RNA and protein levels of E2F1 and RACGAP1 in PC3 cells transiently transfected with *E2F1* si1, si2, or vector, as determined by qRT-PCR and western blot. (J) Relative activity of RACGAP1-luciferase (Luc) reporter 48 h after transient transfection of PC3 cells with an *E2F1* overexpression plasmid for 24 h compared with control cells. The error bars represent the means ± SD of three independent experiments. (K) Full nucleotide sequence of the human *RACGAP1* promoter region. P1 and P2 show the regions of the RACGAP1 promoter explored by the paired primers (left panel). Chromatin immunoprecipitation (ChIP) showed that E2F1 bound at the P1 and P2 loci in PC3 cells (right panel). The error bars represent the means ± SD of three independent experiments. Bar graphs show the statistical analysis of three independent experiments. ***, p < 0.001; **, p < 0.01; *, p < 0.05, p = ns (no significance); *t* test for two groups or ANOVA for more than two groups.

**Figure 5.**
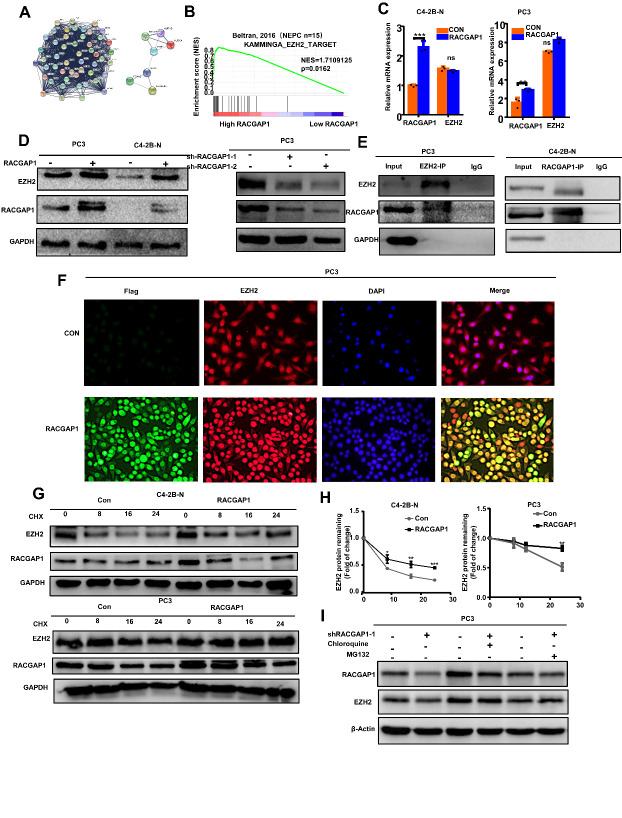
RACGAP1 protein interacts with the EZH2 protein and increases the protein stability of EZH2. (A) Analysis of protein-protein interaction (PPI) information shows that RACGAP1 may directly interact with EZH2. (B) GSEA shows that high *RACGAP1* expression was enriched in the EZH2-TARGET pathway. (C) RNA levels of *RACGAP1* and *EZH2* in NE-like cells treated with *RACGAP1* or empty vector (CON), as determined by qRT-PCR. (D) Western blot was performed to show protein expression of RACGAP1 and E2F1 in NE-like cells following control, *RACGAP1*, *RACGAP1* shRNA1 (sh1), or *RACGAP1* shRNA1 (sh2) transfection. (E) Total cell lysates of NE-like cells were immunoprecipitated with anti-EZH2 or anti-RACGAP1 antibodies and blotted with corresponding antibodies. (F) Representative immunofluorescence images of RACGAP1 and EZH2 protein localization in PC3 cells. (G) C4-2B-N cells and PC3 cells were transiently transfected with CON or *RACGAP1* and supplied with 10 mmol/L cycloheximide (CHX), and then, total cell lysates were collected at 0, 8, 16, and 24 h after treatment. Western blot analysis was used to measure protein levels. (H) Protein expression analysis was used to calculate the half-life of EZH2 protein for C4-2B-N and PC3 cells. (I) Cells transiently transfected with *RACGAP1* knockdown were treated with vehicle (DMSO), chloroquine (50 μm), or MG132 (20 μm) for 12 h. Western blotting was used to detect the protein level of RACGAP1. Bar graphs show the statistical analysis of three independent experiments. ***, p < 0.001; **, p < 0.01; *, p < 0.05, p = ns (no significance); *t* test for two groups or ANOVA for more than two groups.

**Figure 6.**
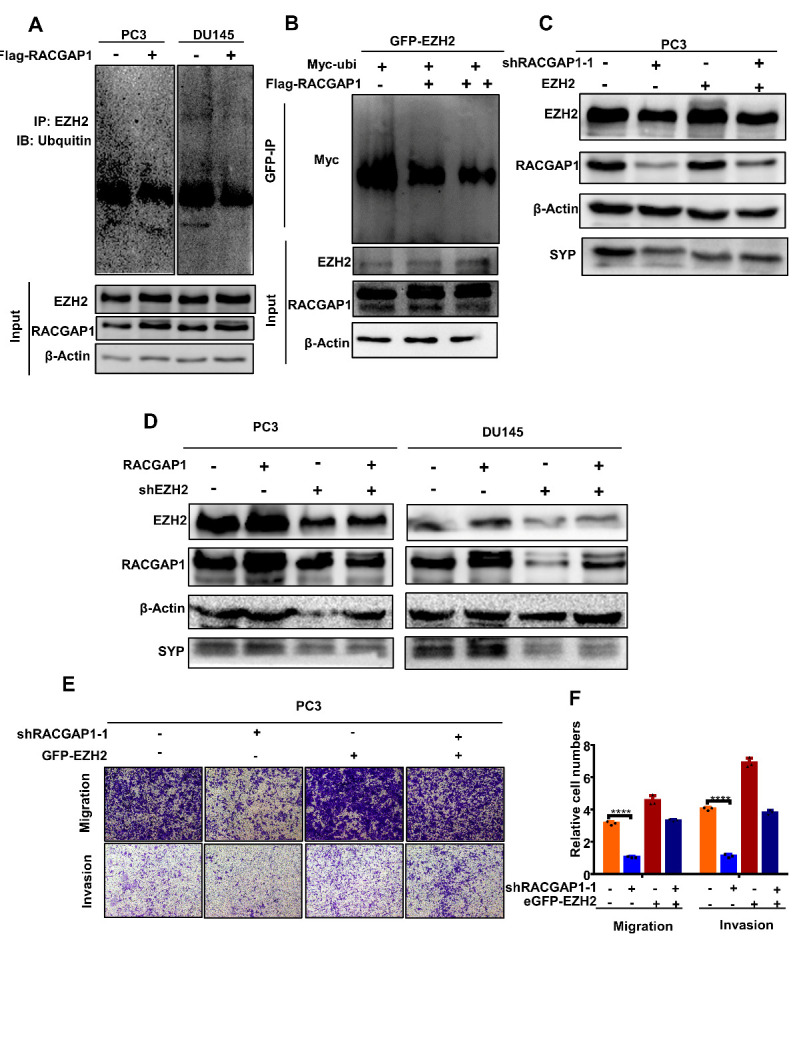
RACGAP1 stabilizes EZH2 protein expression in the ubiquitin-proteasome pathway and affects NED in prostate cancer by regulating EZH2. (A) PC3 and DU145 cells were treated with FLAG-RACGAP1 for 48 h. Total cell lysates were subjected to immunoprecipitation with EZH2 antibody and blotted with an anti-ubiquitin antibody. (B) 293T cells were co-transfected with GFP-EZH2, Myc-ubi, and different doses of FLAG-RACGAP1 (0, 2, 4 µg) for 48 h. Total cell lysates were subjected to immunoprecipitation with a GFP antibody and blotted with an anti-Myc antibody. (C) PC3 cells were transfected for rescue experiments, using PC3 cells with an shRACGAP1 plasmid control (shNC) and *EZH2* expression plasmid control vector (V), cells with the shRACGAP1 plasmid (sh1) and *EZH2* expression plasmid control vector (V), cells with the shRACGAP1 plasmid control (shNC) and *EZH2* expression plasmid, and cells with the shRACGAP1 plasmid (sh1) and *EZH2* expression plasmid. Western blotting was used to detect proteins using the indicated antibodies. We transfected PC3 and DU145 cell lines for rescue experiments, using cells with the *RACGAP1* expression lentivirus control vector (V) and shEZH2 control (shNC), cells with the *RACGAP1* expression lentivirus (RA) and shEZH2 control (shNC), cells with the *RACGAP1* expression lentivirus control vector (V) and shEZH2, and cells with the *RACGAP1* expression lentivirus (RA) and shEZH2. (D) Western blotting was used to detect proteins using the indicated antibodies. (E) Transwell assay for indicated PC3 cells (magnification: 100×). Bar graphs showing the statistical analysis of three independent experiments. ***, p < 0.001, **, p < 0.01, *, p < 0.05, p = ns (no significance); *t* test for two groups or ANOVA for more than two groups.

### E2F1 transcriptionally activates RACGAP1

To study the mechanism of the *RACGAP1* increase in NEPC, we first obtained the promoter sequence of RACGAP1 through the online public database UCSC and searched for it in the PROMO database. At a 5% fault tolerance rate, 54 transcription factors, including E2F1, were predicted to bind to the promoter sequence (Supplementary Fig. 3A, B). We also predicted the upstream transcription factors of *RACGAP1* from the online website GeneCard (https://www.genecards.org/), and the predicted result was 217 genes as possible upstream transcription factors. After overlapping the results, we found that the common predictive transcription factors were E2F1 and YY1 (Fig. 4A). GSEA of the NEPC database showed a significant positive correlation between the expression of *RACGAP1* and the target genes of E2F1 (Fig. 4B). Through the analysis of the GRASSO prostate cancer database[23], we found that the expression of *RACGAP1* was significantly increased in patients with *PTEN*, *RB1*, and *E2F1* gene mutations, suggesting that E2F1 was related to RACGAP1 (Supplementary Fig. 4A-C). A previous study reported that the expression of *E2F1* in NEPC was significantly upregulated, which promoted the neuroendocrine transformation of PCa [31]. We also found that the expression of *E2F1* promoted the progression of prostate cancer (Fig. 4C-G). By knocking down the expression of *E2F1* in PC3 cells, the results showed that the expression of *RACGAP1* decreased significantly after *E2F1* knockdown at both the mRNA and protein levels (Fig. 4H, I). This result suggests that E2F1, as a transcription factor, may be involved in the transcriptional regulation of *RACGAP1*, thus increasing the expression level of *RACGAP1*. To further study the specific mechanism of E2F1 transcriptional regulation of *RACGAP1*, we designed primers aimed at predicting binding sites based on the online database JASPAR (Supplementary Fig. 3B). Dual-luciferase reporter gene and CHIP assays confirmed that E2F1 regulated the expression of *RACGAP1* at the transcriptional level by binding to the promoter region of *RACGAP1* and increasing RACGAP1 transcription (Fig. 4J, K).

### RACGAP1 interacts with and stabilizes the protein expression of EZH2

RACGAP1 can activate STAT3, and EZH2 can also promote the activation of STAT3 in NED [24]. Therefore, we speculated that RACGAP1 and EZH2 are associated with each other. By consulting the literature, we found a significant positive correlation between RACGAP1 and the WNT signaling protein β-catenin, which stabilizes the expression of EZH2 protein [17, 32]. Furthermore, through PPI network analysis and GSEA, we found that there may be an interaction between RACGAP1 and EZH2 (Fig. 5A, B). We analyzed the influence of EZH2 on the stage, grade, and prognosis of PCa using public databases (Supplementary Fig. 5A-E). The results showed that the expression of *EZH2* increased significantly with the progression of PCa. To explore the interaction between *RACGAP1* and *EZH2*, we detected it at both the mRNA and protein levels. The results showed that the protein expression of EZH2 increased with overexpression of *RACGAP1* and decreased when *RACGAP1* was silenced in PC3 cells, but there was no significant difference in *EZH2* mRNA expression (Fig. 5C, D). Because the loss of *RACGAP1* decreased EZH2 protein levels without downregulation of *EZH2* transcript levels, we hypothesized that RACGAP1 regulated EZH2 protein and its function through a posttranslational modification. To detect this possibility, we first used immunoprecipitation experiments. EZH2 was immunoprecipitated from PC3 cell lysates with an anti-EZH2 antibody and tested for RACGAP1 binding by Western blot analysis. RACGAP1 was also immunoprecipitated from C4-2B-N cell lysates with an anti-RACGAP1 antibody and tested for EZH2 binding by Western blot analysis. The results showed that endogenous RACGAP1 co-immunoprecipitated with EZH2 (Fig. 5E). Moreover, we also confirmed that RACGAP1 and EZH2 bound to each other using immunofluorescence co-localization (Fig. 5F). On the basis of the above results, we proposed that RACGAP1 regulated the function of EZH2 through an additional process that may involve the control of EZH2 expression at the posttranslational level. Then, we examined the effect of overexpression of *RACGAP1* on the degradation rate of EZH2 protein by adding 10 µM cycloheximide, an inhibitor of protein synthesis, to PC3 and C4-2B-N cells. The results showed that the degradation rate of EZH2 slowed after overexpression of *RACGAP1* (Fig. 5G, H). These results indicated that RACGAP1 promoted the upregulation of EZH2 by inhibiting its protein degradation.

The autophagy/lysosome pathway and ubiquitin/proteasome pathway are the main pathways that mediate protein degradation in eukaryotic cells [33]. To detect the main pathway for EZH2 degradation, a lysosome inhibitor (chloroquine) and a proteasome inhibitor (MG132) were used on *RACGAP1* knockdown cells. We found that only MG132 reversed the decrease of EZH2 caused by RACGAP1 (Fig. 5I), indicating that RACGAP1 provided the stability of EZH2 protein expression mainly by weakening protein degradation through the ubiquitin/proteasomal pathway. To further verify that RACGAP1 affected the protein stability of EZH2 in the ubiquitin proteasome pathway, we detected the ubiquitin levels of EZH2 after *RACGAP1* overexpression in DU145 and PC3 cells. We found that after upregulation of *RACGAP1*, the ubiquitinated level of EZH2 was lower (Fig. 6A). Different doses of FLAG-RACGAP1, GFP-EZH2, and Myc-ubi plasmids were introduced into 293T cells, and the ubiquitinated level of EZH2 under different doses of RACGAP1 (2 or 4 µg) was further measured (Fig. 6B). The results showed that the ubiquitinated level of EZH2 decreased after overexpression of *RACGAP1* in a dose-dependent manner. These results suggest that RACGAP1 enhanced the protein stability of EZH2 in the ubiquitin-proteasome pathway and promoted the expression of EZH2. Next, western blotting and Transwell rescue experiments showed that RACGAP1 promoted NED in PCa by regulating the expression of EZH2 (Fig. 6C-F).

**Figure 7.**
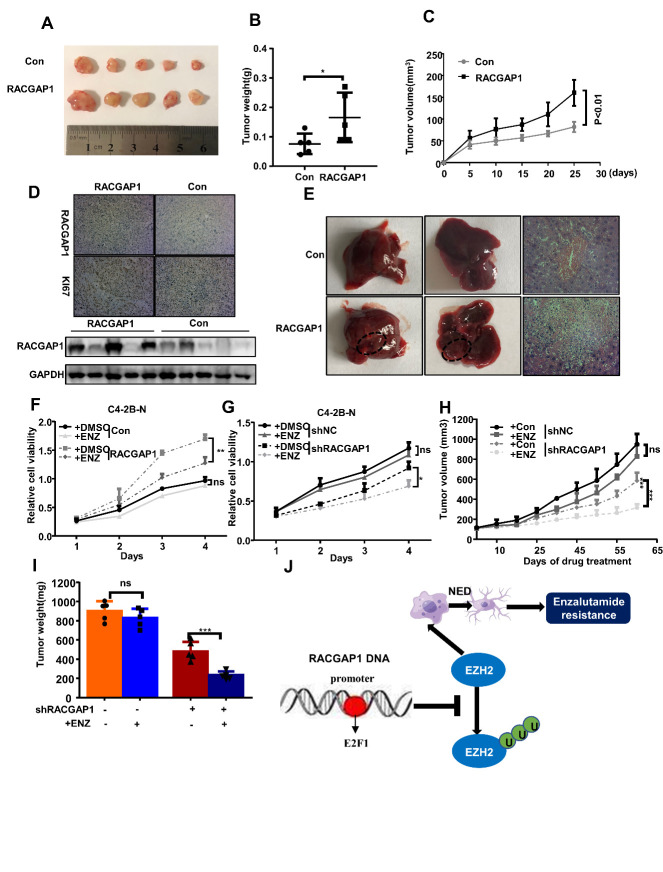
RACGAP1 repressed prostate cancer progression and promoted tumor cell enzalutamide (Enz) resistance. (A, B, C) PC3 cells stably overexpressing RACGAP1 were injected subcutaneously into the armpits of nude mice. Tumor size was detected every 5 days. The data are shown as the mean ± SD. Images of tumors resected from the nude mice. Tumors were weighed after dissection at the end of the experiment. (D) Representative IHC staining images and western blot for RACGAP1 are displayed in the tumor xenografts (IHC magnification: 200×). (E) Hematoxylin-eosin (H&E) staining of the livers of mice in the RACGAP1 overexpression group and control group (magnification: 200×). (F, J) Cell counting kit-8 assays were used to detect the effects of *RACGAP1* upregulation and knockdown on the Enz resistance of C4-2B-R cells. (H) Vector control or enzalutamide (10 mg/kg p.o.) was used to inject nude mice with C4-2B-R cells for stable *RACGAP1* knockdown xenografts for approximately 9 weeks (n = 5). Tumor volumes were detected every 10 days. The data are shown as the mean ± SD. (I) Tumors were weighed after dissection at the end of the experiment. (J) Schematic representations of E2F1/RACGAP1/EZH2-mediated neuroendocrine differentiation in PCa progression. Bar graphs show the statistical analysis of three independent experiments. ***, p < 0.001; **, p < 0.01; *, p < 0.05, p = ns (no significance); *t* test for two groups or ANOVA for more than two groups.

### Effect of RACGAP1 on biological behavior of prostate cancer in vivo

We subcutaneously injected an overexpressing *RACGAP1* lentivirus in PC3 cells into mice. Our results showed that the volume and weight of subcutaneous tumors in the *RACGAP1* overexpression group were significantly higher than those of the control group (Fig. 7A-C). Immunohistochemical staining showed that the expression of Ki67 in the *RACGAP1* overexpression group was significantly higher than that in the control group (Fig. 7D). By injecting PC3 cells overexpressing *RACGAP1* into the tail vein of mice, we observed the effect of overexpression of *RACGAP1* on the formation of metastatic tumors in mice. The changes of metastatic foci were further confirmed by H&E staining. The results showed that evident liver metastasis was found in the mouse metastatic tumor model with overexpression of *RACGAP1*. There was no obvious metastasis in the control group (Fig. 7E). Subsequently, we further examined the effect of *RACGAP1* on enzalutamide resistance of PCa. Our results showed that *RACGAP1* promoted enzalutamide resistance of prostate cancer in vitro and in vivo (Fig. 7F-I, Supplementary Fig. 7A, B). Taken together, we generated a schematic of the progression of enzalutamide resistance-induced NED mediated by the E2F1/RACGGAP1/EZH2 signaling axis and showed that RACCGAP1 is a novel therapeutic target for NEPC (Fig. 7J).

## DISCUSSION

Given the wide application of strong AR inhibitors, the incidence of NEPC is rapidly increasing [34, 35]. At present, the mechanism of NED in PCa is unclear. Through bioinformatics analysis, we found that RACGAP1 was highly expressed in neuroendocrine PCa and that it was positively correlated with several markers of NED and *EZH2*, an important gene in promoting plasticity of neuroendocrine differentiation. Moreover, increased *RACGAP1* expression was significantly associated with several clinicopathologic parameters, such as the Gleason score and tumor stage levels. In addition, high *RACGAP1* expression was correlated with poorer RFS of patients with prostate cancer. Further experimental results showed that RACGAP1 promoted the expression of neuroendocrine differentiation markers. *RACGAP1* was upregulated by E2F1 binding to the *RACGAP1* promoter sequence. Importantly, RACGAP1 maintained the stability of EZH2 protein in the ubiquitin-proteasome pathway and partially reversed enzalutamide resistance. Our results show that RACGAP1 plays an essential role in the progression of PCa NED and enzalutamide resistance.

This work illuminated a previously unappreciated relationship between RACGAP1 activation and EZH2-mediated NED regulation. A previous study found that the expression of *RACGAP1* was significantly increased in NEPC transgenic mice [21]. However, this was found only at the sequencing level, and the mechanism has not been further verified and explored. In addition, *RACGAP1* is overexpressed in NE small cell lung cancer (SCLC) that mirrors NEPC [36]. AURKA is a cell cycle kinase that impedes N-Myc degradation [8, 26, 37]. Interference of the molecular interaction between N-Myc and AURKA is believed to be a treatment method for NEPC [5, 38-41], with promising results. AURKA is directly associated with the expression of *RACGAP1* [17]. In this study, *RACGAP1* expression was screened using related public databases, which confirmed that RACGAP1 is a key and clinically related driving factor promoting the expression of NE markers in progressive PCa, providing a new and powerful basis for treating NEPC. The mechanism by which *RACGAP1* is increased in NEPC is not clear. This study confirmed the transcriptional regulation by E2F1, which promoted *RACGAP1* expression by binding to its promoter sequence. *RACGAP1* is associated with a major genomic characteristic of NEPC: the RB1/E2F1 pathway activation. It provides a reliable theoretical basis for the occurrence and progression mechanism of NEPC. At present, most of the studies on PCa are still at the CRPC stage, while research on the mechanism of NEPC is uncommon. Based on the previous enzalutamide-resistant cell line developed in our laboratory, our team found that RACGAP1 stabilizes the expression of EZH2 in the ubiquitin-proteasome pathway to promote the progression of NED in PCa. RACGAP1 upregulated the expression of EZH2, and EZH2 further stabilized the expression of MYCN and cooperatively inhibited downstream genes suppressed by MYCN [8]. The direct relationship between MYCN/AURKA amplification and abnormal regulation of the RBI/E2F signaling pathway, the two molecular characteristics of NEPC, is not clear. However, our results showed that RACGAP1 connected *MYCN* amplification with the RBI/E2F signal pathway, providing a new direction for the network of NEPC occurrence mechanisms.

RACGAP1 may regulate the downstream factors of the PI3K/AKT signaling pathway in head and neck squamous cell carcinoma [13]. In uterine sarcoma and liver cancer, RACGAP1 activated the STAT3-survivin signaling pathway [14, 15]. There is a direct positive correlation between RACGAP1 and AURKA in gastric cancer [17], and STAT3 and AURKA are all closely related to the neuroendocrine transformation of PCa [8, 26, 37, 42]. In related studies of breast cancer, it was found that high expression of *RACGAP1* was significantly correlated with high histological grade and high NPI [43]. Although the mechanism and function of RACGAP1-induced neuroendocrine transformation in PCa have not been reported, we have reason to believe that RACGAP1 is related to neuroendocrine transformation in PCa. In addition, our previous analysis of the NEPC-related database showed that expression of *RACGAP1* in NEPC was significantly increased. In the TCGA database, we found that *RACGAP1* indicated a poor stage, Gleason score, and survival and prognosis of PCa. Through the UCSC PROMO transcription factor database and predicted transcription factors in GeneCard, as well as GSEA, it was determined that E2F1, a critical factor in NEPC transformation, could be a possible transcription factor upstream of *RACGAP1*. Publicly available data mirrored the correlation between *RACGAP1* and the potential for NE-like disease, as it positively associated with *CHGA* and *SYP* expression. Previous studies have shown a significant positive correlation between RACGAP1 and β-catenin, a WNT signal protein that can stabilize the expression of the EZH2 protein[44]. Using PPI network analysis and GSEA, we found that there may be an interaction between RACGAP1 and EZH2, and protein co-immunoprecipitation revealed that RACGAP1 bound to EZH2. In SCLC and bladder cancer, the RB1-E2F1 signaling pathway upregulated the expression of *EZH2* by binding to the promoter region of *EZH2*. Our results confirmed that E2F1 upregulated the expression of *RACGAP1* in PCa, and RACGAP1 promoted the stability of EZH2 protein, which plays a synergistic role with E2F1 in promoting the expression of EZH2, consistent with the biological characteristics of the tumor. Our experiments show that RACGAP1 is a key regulator involved in the induction of NED in PCa and is associated with E2F1 and EZH2. To summarize, we believe that this project is supported by reliable clinical data analysis of NEPC public databases and related literature results, and therefore, our experimental results are logical and authentic.

RACGAP1 promoted NED of PCa, and the prognosis of PCa is very poor once NED occurs. *RACGAP1* indicates a potential prognosis biomarker related to NED of PCa, which needs further clinical data analysis. EZH2 promoted the epithelial-mesenchymal transformation (EMT) of tumors by binding to the E-cadherin gene (*CDH1*) promoter and catalyzing its H3K27m3 and H3K9m3 methylation [45, 46]. As mentioned earlier, epigenetic reprogramming induced by EZH2 plays an important role in PCa NED. It has been reported that the EMT also promoted the progression to NEPC [47]. Whether RACGAP1 can promote EMT in PCa by increasing EZH2 levels to facilitate neuroendocrine transformation or through other pathways needs further exploration.

In summary, our results explored the positive relationship between *RACGAP1* and PCa stage, Gleason score, and prognosis, demonstrating its potential as a prognostic marker. We confirmed that E2F1 transcriptionally regulated the expression of *RACGAP1* and that RACGAP1 stabilized the expression of EZH2 protein against degradation through the ubiquitin-proteasome pathway. The promotion of RACGAP1 on the biological transition of PCa and NEPC was evaluated using in vitro methods and models of transplanted tumors and metastatic tumors in vivo. Our results showed that RACGAP1 played an important role in promoting PCa NED and the clinical progression of PCa. We verified that the E2F1/RACGAP1/EZH2 signaling pathway was the key target pathway to prevent PCa from developing NED and ADT drug resistance. Our findings provide a new mechanism of neuroendocrine transformation in PCa and a new therapeutic strategy for reversing enzalutamide resistance.

## Supplementary Materials

The Supplementary data can be found online at: www.aginganddisease.org/EN/10.14336/AD.2023.0202.


