# Supplementary material for: Overexpression of RACGAP1 by E2F1 Promotes Neuroendocrine Differentiation of Prostate Cancer by Stabilizing EZH2 Expression
Source: Aging Dis. 2023 Nov 9;14(5):1757–74. doi: 10.14336/AD.2023.0202 (PMC10529746; doi:10.14336/AD.2023.0202)
Supplement: Supplementary file 1 [file AD-14-5-1757-s.pdf]

SUPPLEMENTARY DATA

Overexpression of RACGAP1 by E2F1 Promotes Neuroendocrine Differentiation of Prostate Cancer by Stabilizing EZH2 Expression

Zhengshuai Song, Qi Cao, Bin Guo, Ye Zhao, Xuechao Li, Ning Lou, Chenxi Zhu, Gang Luo, Song Peng, Guohao Li, Ke Chen, Yong Wang, Hailong Ruan, Yonglian Guo

© 2023. Song Z et al. Published online at http://www.aginganddisease.org/EN/10.14336/AD.2023.0202

SUPPLEMENTARY DATA

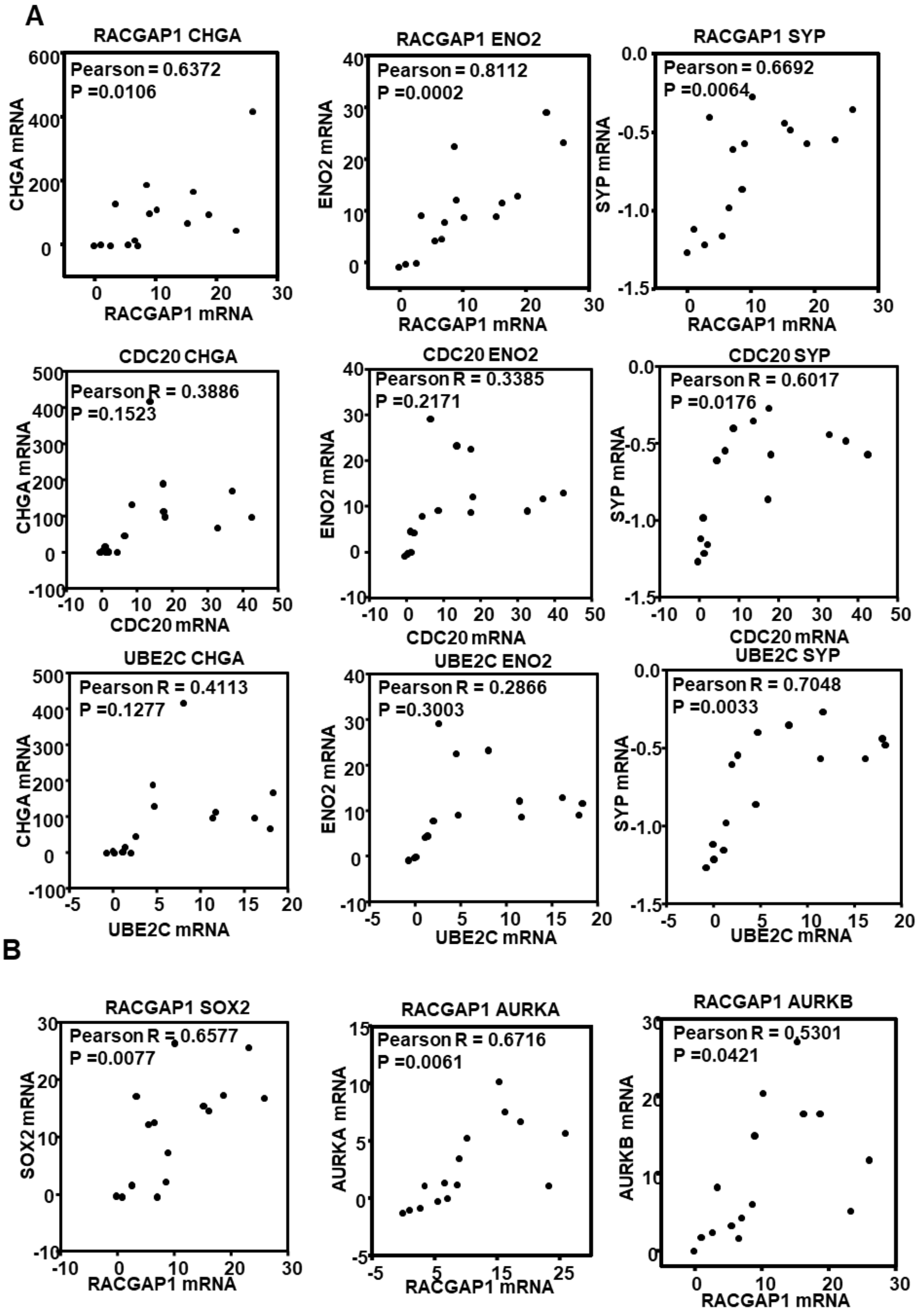

Supplementary Figure 1. There is a significant positive correlation between RACGAP1 and NE markers. (A) RACGAP1, CDC20, and UBE2C correlations with NE markers were determined by Spearman rank correlation in NEPC datasets. (B), The correlations between RACGAP1 and other NED important regulatory factors. The results are shown as the mean ± SD. *p < 0.05; **p < 0.01, ***p < 0.001.

© 2023. Song Z et al. Published online at http://www.aginganddisease.org/EN/10.14336/AD.2023.0202

SUPPLEMENTARY DATA

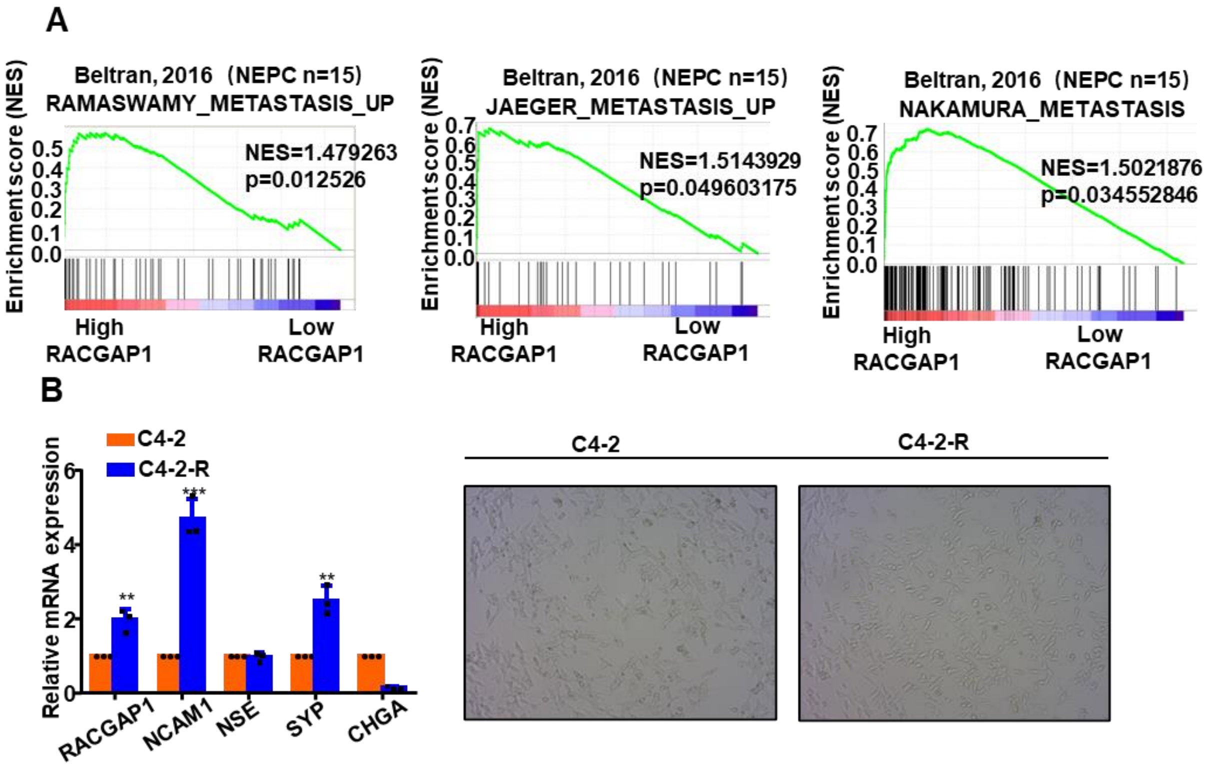

Supplementary Figure 2. Gene set enrichment analysis (GSEA) of RACGAP1. (A) 3 representative tumor Metastasis Pathway enriched in NEPC with highly expressed RACGAP1. (B) Relative mRNA expression of RACGAP1, NE markers and Morphological changes of C4-2-R compared with C4-2. Bar graphs showing the statistical analysis of three independent experiments. ***, p < 0.001; **, p < 0.01; *, p < 0.05, p = ns (no significance). t-test for two groups or ANOVA for more than two groups.

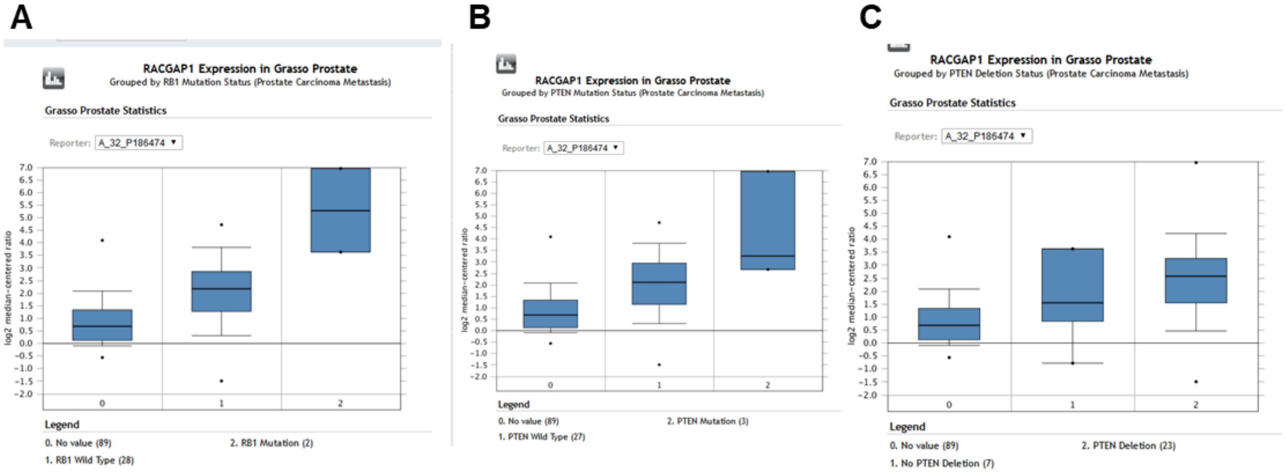

Supplementary Figure 3. Expression of RACGAP1 in different groups of prostate cancer. (A, B, C) Change of RACGAP1 expression value grouped according to RB1 Mutation, PTEN Mutation and PTEN Deletion Status. *p < 0.05; **p < 0.01, ***p < 0.001.

© 2023. Song Z et al. Published online at http://www.aginganddisease.org/EN/10.14336/AD.2023.0202

SUPPLEMENTARY DATA

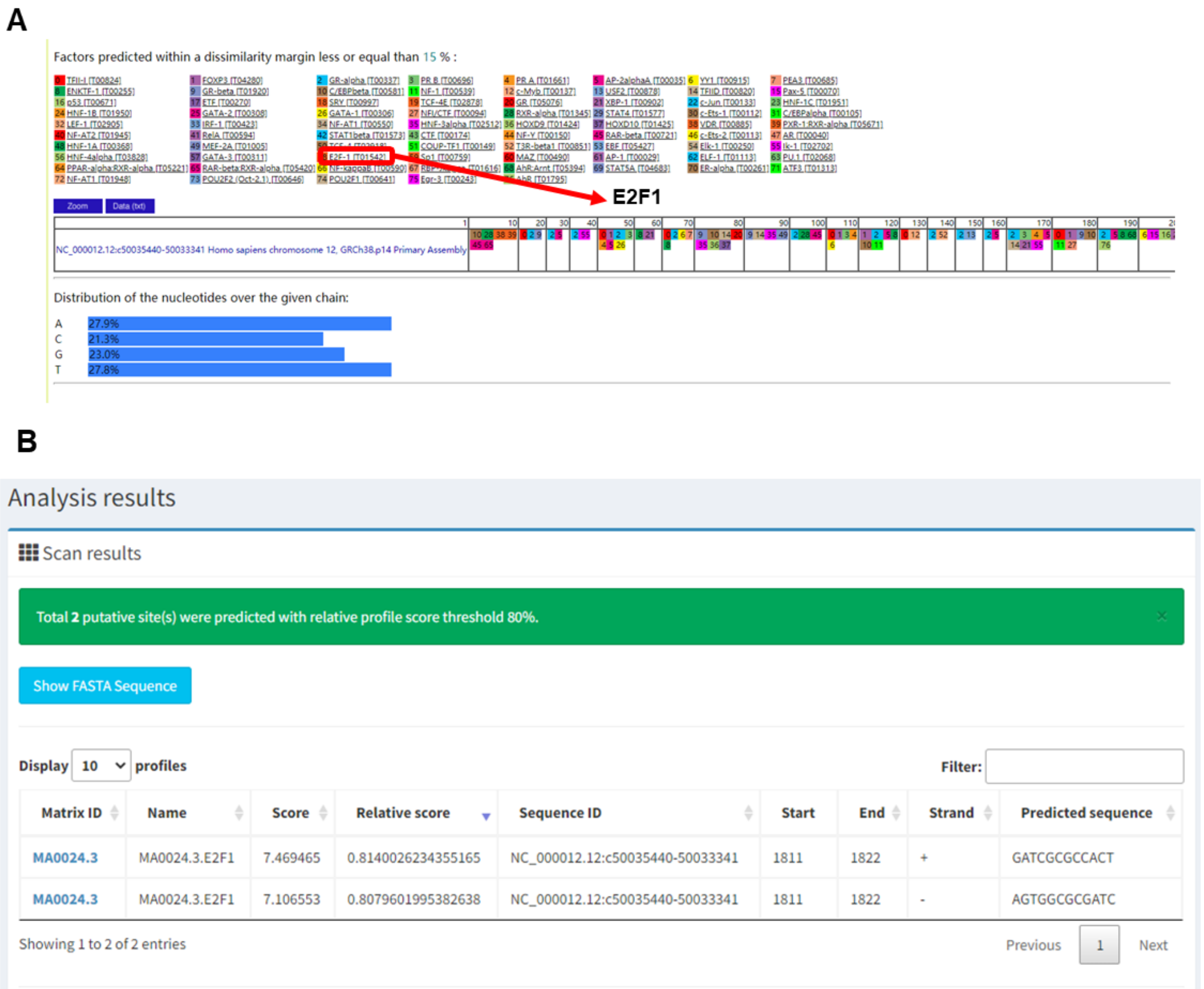

Supplementary Figure 4. Possible transcription factors and corresponding binding sequences of RACGAP1. (A) Through PROMO online transcription factor prediction software, the RACGAP1 promoter sequence was placed to retrieve possible transcription factors. (B) Binding sequences of E2F1 and RACGAP1 were predicted by JASPAR.

© 2023. Song Z et al. Published online at http://www.aginganddisease.org/EN/10.14336/AD.2023.0202

SUPPLEMENTARY DATA

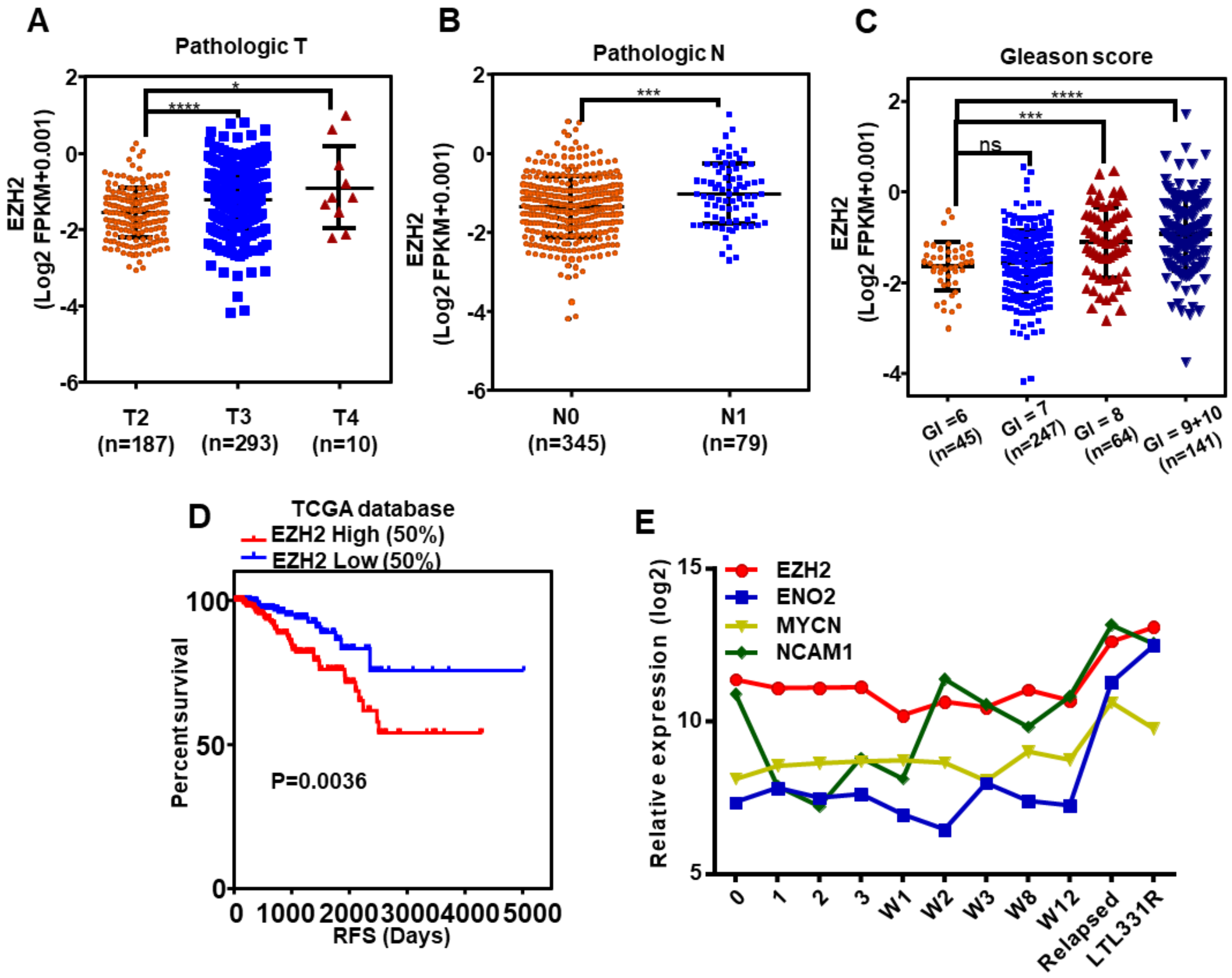

Supplementary Figure 5. Relationship between EZH2 and clinicopathological parameters of prostate cancer. Changes of EZH2 expression in different clinicopathological parameters downloaded from the TCGA-PRAD: (A) Pathologic T stage, (B) Pathologic N stage, (C) Gleason score. (D) Patients with high expression of EHZ2 have shorter recurrence-free survival. (E) EZH2 and NE markers were highly expression in NEPC tumor samples (adenocarcinoma pre-castration days 0; post-castration days 1–3; weeks 1–3, 8, and 12; and post-NEPC development Relapsed and LTL331R. Data downloaded from GSE59986). TCGA-PRAD, The Cancer Genome Atlas Prostate Cancer. ***, p<0.001, **, p<0.01, *, p<0.05.

© 2023. Song Z et al. Published online at http://www.aginganddisease.org/EN/10.14336/AD.2023.0202

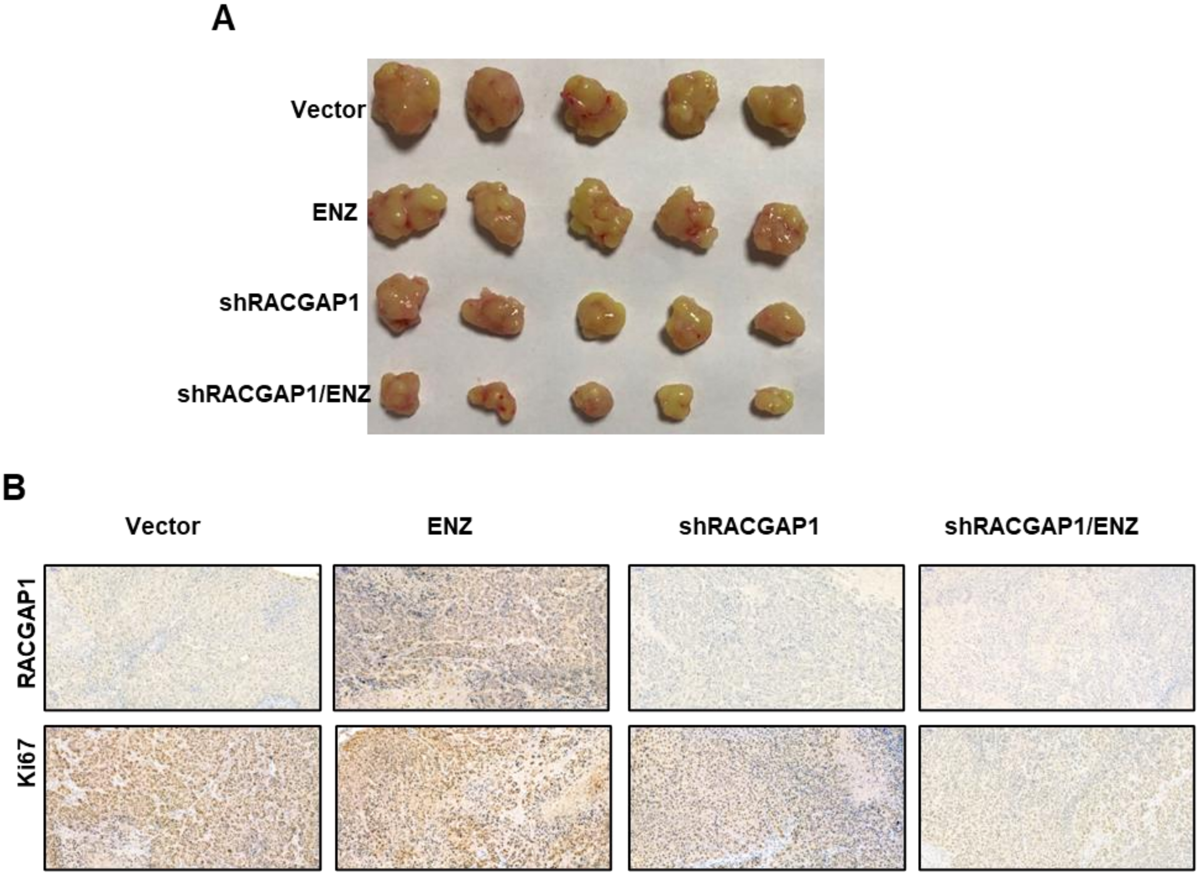

Supplementary Figure 6. Experiment of tumor xenografts in mice. (A) Images of tumors dissected from the mice in different groups. (B) Immunohistochemical (IHC) staining for the marker of tumor malignancy (KI67) RACGAP1 and RACGAP1 in the tumor xenografts.

© 2023. Song Z et al. Published online at http://www.aginganddisease.org/EN/10.14336/AD.2023.0202
